# Supplementary material for: Integrating cell morphology with gene expression and chemical structure to aid mitochondrial toxicity detection
Source: Commun Biol. 2022 Aug 23;5:858. doi: 10.1038/s42003-022-03763-5 (PMC9399120; doi:10.1038/s42003-022-03763-5)
Supplement: Supplementary file 3 — Description of Additional Supplementary Files [file 42003_2022_3763_MOESM3_ESM.pdf]

# Description of Additional Supplementary Files

**File name:** Supplementary Data 1
**Description: Comparison our models with models previously published in mitochondrial toxicity. Although the external test sets differ and results are not directly comparable, our models achieve comparable balanced accuracy to Hemmerich et al while increasing sensitivityto mitochondrial toxic compounds compared to some dedicated high content imaging assays**

**File name:** Supplementary Data 2
**Description: Mitochondrial toxicity, MOA and effect of on mitochondiral for compounds outlier to majority of compounds in morphology space**

**File name:** Supplementary Data 3
**Description: Computational significance and biological implication in mitochondrial toxicity of Cell Painting features most correlated to 10 Gene Expression features that are related to mitochondiral toxicity mechanisms.**

**File name:** Supplementary Data 4
**Description: Positive predicitive values and F1 scores of few individual Cell Painting features for 486 compounds (85 mitotoxic). We translate the computational significance of Cell Painting features to a biological implication mitochondrial toxicity, mainly for features related to edge intensity of cells (possibly related to integrity of cell wall), radial distribution and intensity in mitochondria (related to mitochondrial death) and granularity features (related to cell death and amount of information contained in cellular images).**

**File name:** Supplementary Data 5
**Description: Positive predicitive values and F1 scores of few individual Gene Expression features for 486 compounds (85 mitotoxic). We translate the computational significance of to a biological implication mitochondrial toxicity, mainly for features related to unfolded protein response and calcium channels (possibly related to ER stress) and plasma membrane (related to membrane depolarisation)**

**File name:** Supplementary Data 6
**Description: Evaluation metrics of the seven models (a) median of repeated nested cross validations and (b) external test set. The best performing model according to the metric is highlighted in bold. Early-stage fusion and Late-stage fusion models have higher F1 score for compounds exhibiting mitochondrial toxicity and extrapolate well into new chemical space in external test sets compared to models using Morgan fingerprints. CP: Cell Painting; GE: Gene Expression; NCV: Nested Cross Validation; ET: External Test**

**File name:** Supplementary Data 7
**Description: Some external test set mitochondrial toxicants and their mechanism of action from literature**

**File name:** Supplementary Data 8
**Description: Predictions from models for inconclusive compounds in Tox21 assay and related literature on mitochondrial toxicity**

**File name:** Supplementary Data 9
**Description: Summary of performance of our models for inconclusive compounds in Tox21 (10 toxic and 4 non-toxic from literature)**

**File name:** Supplementary Data 10
**Description: Comparison of results from different Feature Selection methods. CP: Cell Painting, GE: Gene Expression**

**File name:** Supplementary Data 11
**Description: Training Dataset used for Nested Cross Validation**

**File name:** Supplementary Data 12
**Description: External Test Dataset used for External Validation**
